# An unusual relationship between the axillary artery and brachial plexus in a cross-breed dog cadaver: a proposal for revising the terminology for nerve loops

**DOI:** 10.1186/s12917-024-04041-8

**Published:** 2024-05-11

**Authors:** Younes Kamali

**Affiliations:** Department of Basic Sciences, Faculty of Veterinary Medicine, Ferdowsi University of Mashhad, Mashhad, Iran

**Keywords:** Axillary artery, Axilla, Nerve loop, Dog

## Abstract

In the left axilla of a formalin-embalmed adult female cross-breed dog, an unusual course of the axillary artery in relation to the brachial plexus was noted. A part of the axillary artery after the origin of the subscapular artery coursed through the loop formed by the contributions of the caudal pectoral and lateral thoracic nerves and then between the median and ulnar nerves. Thus, the common trunk for the latter two nerves was missing. Instead, in the proximal brachium, they communicated with each other in both directions. A communicating branch between the cranial and caudal pectoral nerves forming a nerve loop, *ansa pectoralis* lacked the axillary artery and was instead traversed by the subscapular artery. This is a variation in the relationship between the axillary artery and brachial plexus in the domestic dog and has not been reported in the literature yet. The axillary artery entrapped by the contributions of the caudal and lateral thoracic nerves may be considered as a risk factor for the neuroarterial compressions with non-specific signs and should be taken into account both in surgery and imaging.

## Background

In domestic dogs, the median and ulnar nerves depart the brachial plexus together as a common trunk [1, 2]. The musculocutaneous and cranial pectoral nerves take inputs predominantly from the ventral branch of C7, while the common trunk of the median-ulnar nerves as well as the caudal pectoral nerves receive inputs primarily from C8 and T1 (T2) [2, 3].

Regarding the relationship of the axillary artery with the brachial plexus, the axillary artery runs distally between the musculocutaneous nerve cranially and the common trunk of the median and ulnar nerves caudally. In this relation, the radial and caudal pectoral nerves are lateral and caudal to the artery, respectively [1]. Variations in the branching pattern of the brachial plexus depending on its position in relation to the axillary artery have been reported in several primate studies [4, 5]. In the domestic dog, the axillary artery passes between the continuation of the C7 and C8 ventral branches, which is consistent with what has been described for most primates [4, 5]. Knowledge of the anatomical variants of the axillary artery in relation to the brachial plexus can be helpful in explaining atypical clinical symptoms that may appear following certain vigorous movements [6].

In dog anatomy texts, there are no descriptions of the communicating branches forming nerve loops in the course of the axillary artery. The traces of such communicating branches extending to the caudal pectoral nerves can only be found in the illustrations of Miller’s *Anatomy* [1]. With a comparison of the nerve loops in other studies on carnivorans and those in the current case, it seems that the terminology needs some revisions to address what they mean exactly [7–9].The aim of the present case report is to introduce an unusual anatomical relationship between the axillary artery and brachial plexus identified in an adult female cross-breed dog cadaver and to propose a revision of nomenclature on the nerve loops of the brachial plexus.

## Case presentation

During the dissection of the left axilla of a formalin-embalmed adult female cross-breed dog for educational purposes, the following rare neuroarterial relationship was identified. The information of the axillary artery in relation to the brachial plexus in the right axilla was inadvertently destroyed by undergraduate veterinary students dissecting the intrinsic musculature. To investigate the relationship of the axillary artery and the nerve loops of the brachial plexus in more detail, the present specimen was compared with three previously dissected specimens in our dissection room over three years. The dogs were obtained from the kennel of the School of Veterinary Medicine, Shiraz University. They were euthanized by the injection of IV sodium pentobarbital (85 mg/kg) based on the recommendations of the *AVMA Guidelines for the Euthanasia of Animals* (2020 Edition). For a better visualization of the arteries, red-colored gelatine was injected into the cannulated common carotid artery.

**Fig. 1:**
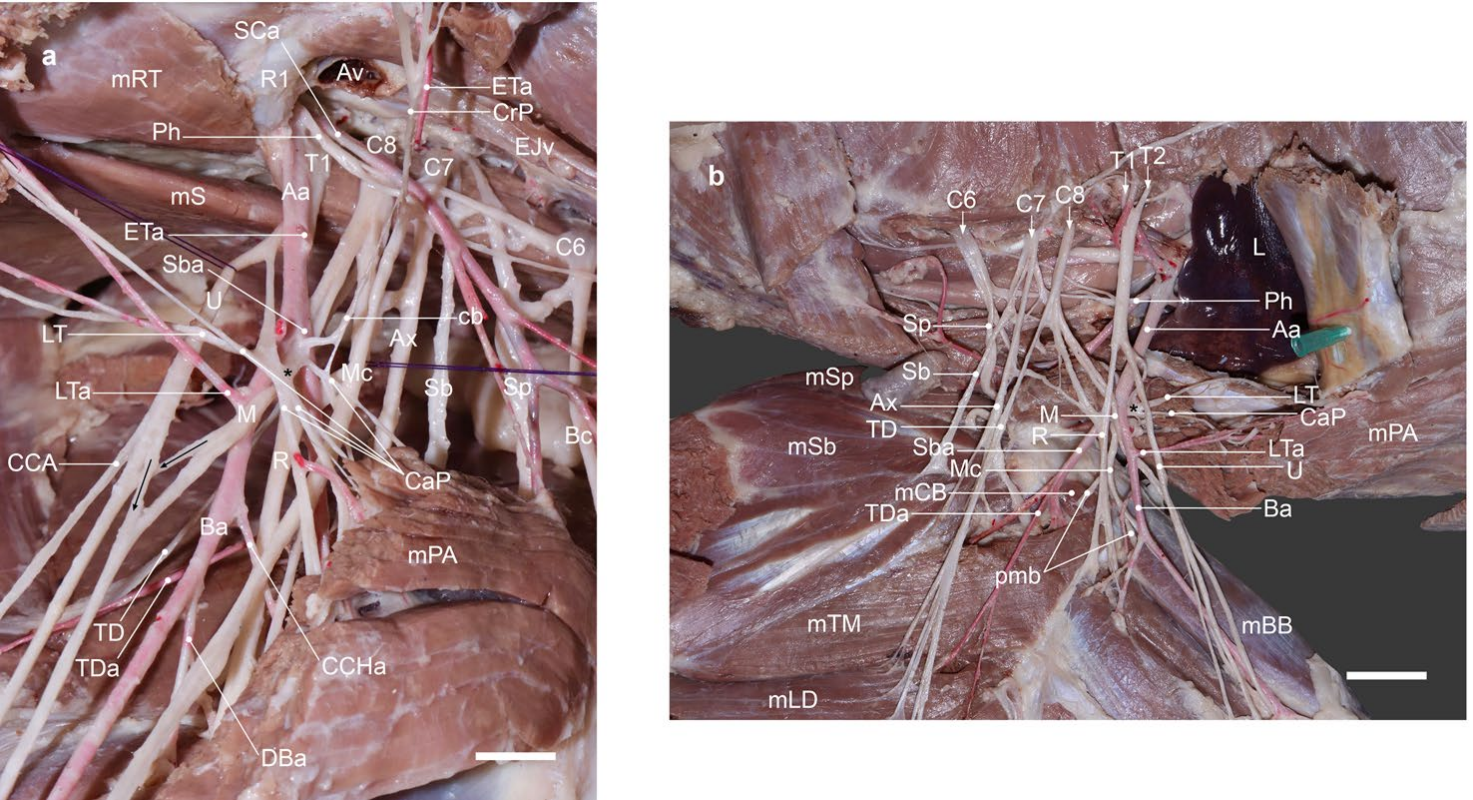
(**a**) Ventromedial and (**b**) dorsolateral views of the left brachial plexus in an adult female cross-breed dog. C6-8, ventral branches of cervical spinal nerves; T1 & T2, ventral branches of thoracic spinal nerves that T2 in the ventromedial view is not exposed. The axillary artery (Aa) after the origin of the subscapular artery (Sba) passing through the loop (*) formed by the contributions to the caudal pectoral (CaP) and lateral thoracic (LT) nerves, and then between the median (M) and ulnar (U) nerves. Av, axillary vein; Ax, axillary nerve; Ba, brachial artery; black arrows, communications between the M and U; Bc, nerve to brachiocephalicus m. (mBC); cb, communicating branch between the cranial pectoral nerve (CrP) and the loop (*) passing medial to the Sba; CCA, caudal cutaneous antebrachial nerve; CCHa, cranial circumflex humeral artery; DBa, deep brachial artery; EJv, external jugular vein; ETa, external thoracic artery; L, lung; LTa, lateral thoracic artery; Mc, musculocutaneous nerve; mBB, biceps brachii m.; mCB, coracobrachialis m.; mLD, latissimus dorsi m.; mPA, pectoralis ascendens m.; mRT, rectus thoracis m.; mS, scalenus m; mSb, subscapularis m.; mSp, supraspinatus m.; mTB, triceps brachii m.; mTM, teres major m.; Ph, phrenic nerve; pmb, proximal muscular branches; R, radial nerve; R1, rib 1; Sb, subscapular nerve; SCa, superficial cervical artery; Sp, suprascapular nerve; TD, thoracodorsal nerve; TDa, thoracodorsal artery. Scale bar: 2 cm

In the left axilla, an unusual course of the axillary artery was noted. A part of the axillary artery after the origin of the subscapular artery coursed through the loop formed by the contributions of the caudal pectoral and lateral thoracic nerves and then between the median and ulnar nerves. Therefore, the common trunk for the median and ulnar nerves was missing. Instead, in the proximal brachium, they connected with each other through two communicating branches running in both directions (Fig. 1a). The median nerve took its origin from the C8 branch proximally with a contribution from T1 and T2 through the communicating branch from the ulnar nerve distally (Fig. 1a & b). At first, it descended over the lateral aspect of the origin of the subscapular artery, together with a branch contributing to the caudal pectoral nerves. Then, it crossed obliquely cranial to the axillary artery from lateral to medial aspects and passed distad to lie caudal to the brachial artery. The subscapular artery arose from the axillary artery more proximally than usual, about midway between the origins of the external thoracic and lateral thoracic arteries (Fig. 1a).

The contributions to the formation of the caudal pectoral and lateral thoracic nerves united ventral to the axillary artery, forming a loop around that artery just distal to the origin of the subscapular artery (Fig. 1a & b). One branch arose with the median nerve from C8, accompanied in its course by that nerve to the subscapular artery origin, where it joined the other branch arising with the ulnar nerve from T1 and T2. Several branches to the deep pectoral muscle and the lateral thoracic nerve arose from the loop connecting the two branches. In addition, a branch with a small contribution from the musculocutaneous nerve to the coracobrachialis muscle also originated from this loop. The loop also formed a slender communicating branch to the cranial pectoral nerve.

**Fig. 2:**
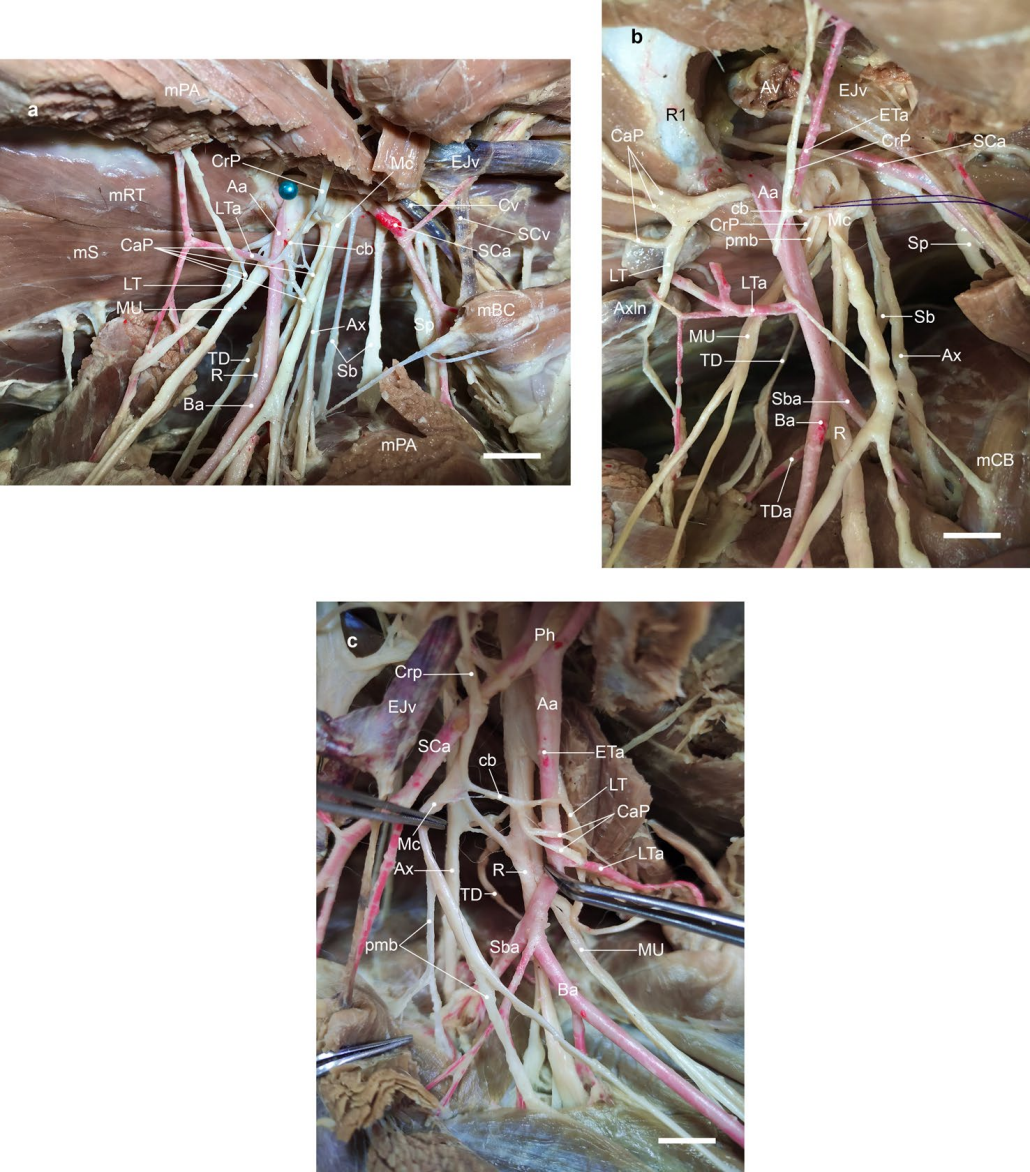
Ventromedial view of three dissections showing three types of communicating branch (cb) contributes to the formation of a nerve pectoral loop surrounding the axillary artery (Aa). (**a**) the cb between the cranial pectoral nerve (CrP) and common trunk of median and ulnar nerves (MU) forming a nerve loop just distal to the origin of the lateral thoracic artery (LTa); (**b**) the cb between the CrP (arising together with the proximal muscular branch to the coracobrachialis muscle from the C8 and T1) and musculocutaneous nerve (Mc) forming a nerve loop just distal to the origin of the external thoracic artery (ETa); (**c**) the cb between the Mc and the caudal pectoral (CaP) and lateral thoracic (LT) nerves forming a nerve loop between the origins of the external thoracic artery (ETa) and lateral thoracic artery (LTa). Notice the origin of the CrP in (**b**). Axln, proper axillary lymph node; Cv, cephalic vein; SCv, superficial cervical vein. Scale bar: 2 cm

Dissections on the brachial plexus of three other cross-breed dogs in our dissection room over three years revealed the presence of a similar communicating branch with at least one group of pectoral nerves (Fig. 2a-c).

## Discussion

In the present case, the common trunk of the median and ulnar nerves was absent. After giving off the subscapular artery, the axillary artery passed through the loop formed by the contributions of the caudal pectoral and lateral thoracic nerves and then between the median and ulnar nerves. This is an unusual variation in the relationship between the axillary artery and brachial plexus in the domestic dog and has not been reported in the literature so far. In canids, the ventral branches of C8 and T1 (T2) usually join together closely to contribute to the formation of the median-ulnar common trunk as well as the caudal pectoral and lateral thoracic nerves [1, 10]. Thus, the axillary artery runs distally between the musculocutaneous nerve arising from C7 cranially and the median-ulnar common trunk arising from C8 caudally. The radial as well as the lateral thoracic and caudal pectoral nerves are also situated lateral and caudal to that artery, respectively [1, 3]. A similar positional relationship between the artery and brachial plexus has also been described in man and other primates [4]. Most frequently, the axillary artery in man runs ventrally through the brachial plexus between the C7 and C8 spinal nerves [4, 5]. With a lower frequency, other variations in their relationship have also been reported [4].

In the left side of the case presented here, the axillary artery anomalously penetrated the brachial plexus more caudally and laterally between the continuations of the ventral branches of C8 and T1. In other words, during morphogenesis, although the constituents of the brachial plexus were developed normally, the deviation in the course of the axillary artery was possibly responsible for this variation. Therefore, the arrangement of the brachial plexus could have followed a normal pattern if the axillary artery had coursed cranially to the nerve loop formed by the contributions of the caudal pectoral and lateral thoracic nerves rather than through it.

As noted in dissections of the brachial plexus of three other cross-breed dogs, a communicating branch may be present with at least one group of pectoral nerves (Fig. 2a-c). The formed nerve loop traversed by a portion of the axillary artery has been called ‘*ansa pectoralis*’ based on the nomenclature presented by the authors of several studies on carnivorans though it has not been used in the NAV [8, 9, 11, 12]. Put simply, if the pectoral loop is present, the axillary artery passes through it. In the study of Nakamura et al. on both axilla of 10 beagle dogs [13], several types of communicating branches were described. In type 1, the communicating branch of the cranial pectoral nerve was attached to the caudal pectoral nerve (5 axilla), to the ventral branch of C8 (3 axilla), and to both the caudal pectoral nerve and the ventral branch of C8 (2 axilla). In addition, although the illustrations of Miller’s *Anatomy* (Figs. 17 − 2, 17 − 11, 17 − 13) show the presence of a communicating branch to the caudal pectoral nerves on the course of the axillary artery, the text lacks any description in this regard [1].

The communicating branch between the cranial pectoral nerve and caudal pectoral nerves in the present case extended cranially to the axillary artery rather than medially and the formed nerve loop was traversed by the subscapular artery. Instead, the axillary artery was entrapped medially by the nerve loop formed by the contributions of the caudal pectoral and lateral thoracic nerves due to a caudal and lateral deviation in its course, as already mentioned. The latter nerve loop can be normally observed in dogs. However, the passage of the axillary artery through it gives the impression that a new loop has been formed. This is comparable to a nerve loop without any nomenclature formed by the cranial pectoral nerves around the axillary artery in ruminants before its passage between the musculocutaneous and median nerves [14].

From clinical point of view, an unusual course of the axillary artery through a nerve loop and then between the median and ulnar nerves in the case described here may cause a potential disruption in blood supply and ischemia, as suggested by other types of nerve loops in human medicine and the author’s previous report in the domestic dog [6, 7]. On the contrary, the result of such entrapment of the artery due to its natural pulsation can stimulate the corresponding nerve in some specific movements of the shoulder and arms, leading to clinical symptoms of unknown origin [6].

Finally, a revision to the terminology of the nerve loops of the brachial plexus was prompted by this case since the current terminology does not exactly address the structural nature of these loops, especially when their variants are encountered. As a communicating branch of the musculocutaneous nerve to the median nerve, the term *ansa axillaris* has been proposed by some authors working on carnivorans including the domestic dog [7–9]. Nevertheless, in the study of Davis on some arctoid carnivores, the presence of such communication has been defined as *ansa mediana* in the Ailurid *Ailurus fulgens*, the Ursid *Ursus americanus*, and procyonids *Procyon lotor* and *Potos flavus* [12].

To avoid any contradictions in the terminology of the nerve loops of the brachial plexus each of which is traversed by part of the axillary artery, it is proposed that the ansa axillaris in Ungulata be complemented as ***ansa axillaris mediana***. For this term, one end of the communicating branch must be attached to the median nerve. In addition, to better address the pectoral nerve loop traversed by the axillary artery, the term ***ansa axillaris pectoralis*** seems to be more suitable than the term *ansa pectoralis*. The problem of the latter term is that it does not refer to its relation to the axillary artery. Hence, it is not comparable to the term *ansa cervicalis* which lacks an artery. The term *ansa pectoralis* seems to be more appropriate for the nerve loop of the present case through which the subscapular artery passes rather than the axillary artery.

## Conclusions

The unusual course of the axillary artery through the contributions of the caudal pectoral and lateral thoracic nerves may be considered as a risk factor for the neuroarterial compressions with non-specific signs and should be taken into account both in surgery and imaging. This variation can be of high importance from the embryogenic and phylogenetic perspectives.

## Data Availability

The datasets generated and/or analyzed during the current study are available from the corresponding author on request.

## References

[1] Evans HE, De Lahunta A. Miller’s anatomy of the dog-E-Book. Elsevier health sciences; 2013.

[2] Getty R, Sisson S (1975). Scission and Grossman’s the anatomy of the domestic animals.

[3] Allam MW, Lee DG, Nulsen FE, Fortune E (1952). The anatomy of the brachial plexus of the dog. Anat Rec.

[4] Kikuchi Y, Oishi M, Shimizu D (2011). Morphology of brachial plexus and axillary artery in bonobo (Pan paniscus). Anat Histol Embryol.

[5] Johnson EO, Vekris M, Demesticha T, Soucacos PN (2010). Neuroanatomy of the brachial plexus: normal and variant anatomy of its formation. Surg Radiologic Anatomy: SRA.

[6] Saeed M, Rufai AA (2003). Median and musculocutaneous nerves: variant formation and distribution. Clin Anatomy: Official J Am Association Clin Anatomists Br Association Clin Anatomists.

[7] Kamali Y (2022). Aberrant arrangement of the musculocutaneous and median nerves in the thoracic limbs of a mixed-breed dog cadaver. Anat Histol Embryol.

[8] Velez Garcia JF, de Carvalho Barros RA, Miglino MA. Origin and distribution of the Brachial Plexus in two procyonids (Procyon cancrivorus and Nasua nasua, Carnivora). Anim (Basel) 2023, 13(2). 10.3390/ani13020210 PMC9854546 36670750

[9] Enciso García LM, Vélez García JF (2022). Origin and distribution of the brachial plexus in kinkajou (Potos flavus–schreber, 1774). Anat Histol Embryol.

[10] de Souza Junior P, da, Cruz de Carvalho N, de Mattos K, Abidu Figueiredo M, Luiz Quagliatto Santos A. Brachial Plexus in the Pampas Fox (Lycalopex gymnocercus): a Descriptive and Comparative Analysis. *Anat Rec (Hoboken)* 2017, 300(3):537–548. 10.1002/ar.23509 27788289

[11] NAV NAV: The International Committee on Veterinary Gross Anatomical Nomenclature. *Published by the Editorial Committee Hannover (Germany), Columbia, MO (USA), Ghent (Belgium), Sapporo (Japan), 6th edition (Revised version)* 2017.

[12] Davis DD (1964). The giant panda: a morphological study of evolutionary mechanisms. Fieldiana (Zoology Memoirs).

[13] Nakamura M, Tomizawa N, Tohyama K, Hara S (2004). Morphological variations in brachial plexus of beagle dogs: evaluation of utility as sources of allogeneic nerve grafts. J Vet Med Sci.

[14] Sisson S, Grossman JD, Getty R. The anatomy of the domestic animals. Volume 1. IWB Saunders Comp, Philadelphia–London–Toronto; 1975.

